# Characterization of a New Hsp110 Inhibitor as a Potential Antifungal

**DOI:** 10.3390/jof10110732

**Published:** 2024-10-23

**Authors:** Cancan Sun, Yi Li, Justin M. Kidd, Jizhong Han, Liangliang Ding, Aaron E. May, Lei Zhou, Qinglian Liu

**Affiliations:** 1 Department of Physiology and Biophysics, School of Medicine, Virginia Commonwealth University, Richmond, VA 23298, USA; 2 Institute of Molecular Physiology, Shenzhen Bay Laboratory, Shenzhen 518107, China; 3 Department of Physiology and Pathophysiology, School of Basic Medical Sciences, Capital Medical University, Beijing 100069, China; 4 Department of Medicinal Chemistry, School of Pharmacy, Virginia Commonwealth University, Richmond, VA 23298, USA

**Keywords:** fungal infections, Hsp110, molecular chaperones, *Candida albicans*, *Candida auris*, *Cryptococcus neoformans*, pharmacokinetic

## Abstract

Fungal infections present a significant global health challenge, prompting ongoing research to discover innovative antifungal agents. The 110 kDa heat shock proteins (Hsp110s) are molecular chaperones essential for maintaining cellular protein homeostasis in eukaryotes. Fungal Hsp110s have emerged as a promising target for innovative antifungal strategies. Notably, 2H stands out as a promising candidate in the endeavor to target Hsp110s and combat fungal infections. Our study reveals that 2H exhibits broad-spectrum antifungal activity, effectively disrupting the in vitro chaperone activity of Hsp110 from *Candida auris* and inhibiting the growth of *Cryptococcus neoformans*. Pharmacokinetic analysis indicates that oral administration of 2H may offer enhanced efficacy compared to intravenous delivery, emphasizing the importance of optimizing the AUC/MIC ratio for advancing its clinical therapy.

## 1. Introduction

As a formidable global health challenge, fungal infections exert a profound impact on a substantial population worldwide [1,2,3,4,5,6,7,8,9,10]. Millions grapple with these infections, and the peril they pose can be life-threatening for the immunocompromised. The World Health Organization (WHO) has identified a group of fungi within a “critical priority” category [11], including *Candida* species and *Cryptococcus neoformans*. *Candida* is one of the leading causes of hospital-acquired infections and can cause disseminated bloodstream infections (candidemia) with mortality rates over 40% [1,2,12]. As an opportunistic pathogen, *Candida* induces various infections collectively termed candidiasis [13], such as oral thrush, vaginal yeast infections, and candidemia. While some are commensal, a number of *Candida* species (e.g., *albicans*, *glabrata*, and *auris*) can cause serious infections for critically ill and/or immunocompromised individuals, such as patients with AIDS, cancers, organ transplants, or implants [3,4,5,6,7,8,9,14,15]. As the most common fungal pathogen, *C. albicans* is responsible for about half of all cases of candidiasis in humans [9,10]. *C. auris*, an emerging multidrug-resistant yeast [16,17], poses a global health threat, causing invasive infections, particularly candidemia, associated with elevated mortality rates. Similar to *Candida*, *C. neoformans*, an opportunistic pathogen that causes cryptococcosis, predominantly affects immunocompromised individuals, leading to severe respiratory and central nervous system complications [18,19].

In comparison with the wide variety of antibacterial agents, treatment for fungal infections such as candidiasis is limited to only five classes of antifungal drugs: azoles, polyenes, echinocandins, allylamines, and triterpenoids [20,21,22,23,24,25,26]. These antifungals primarily target to the cell wall and membrane. In addition, the pyrimidine analog 5-flucytosine [27], which inhibits RNA synthesis, is also used as an antifungal. Moreover, many broad-spectrum antifungals, such as polyenes, have serious side effects that pose major concerns [28]. Additionally, there has been a dramatic rise in resistance to these drugs [2,20,21,22]. The recently identified *C. auris* is especially attracting widespread attention due to its multiple drug resistance [16,17].

Developing antifungals has proven to be challenging. Only two new classes of antifungals, echinocandins and triterpenoids, have been approved for therapeutic use over the last two decades [22,24,29]. This is, in part, due to the many evolutionarily conserved metabolic pathways shared between fungi and humans, making selectivity more difficult to achieve than with antibacterials [30]. Another confounding factor is the limited understanding of basic fungal biology, leading to a lack of known antifungal targets and mechanisms [31,32]. Thus, there is an urgent need to discover potent therapeutic options with novel modes of action to treat various fungal infections.

Heat shock proteins 110 kDa (Hsp110s) have emerged as a promising target against fungal infections [33]. Hsp110s form a unique class of molecular chaperones ubiquitously present in the cytosol of eukaryotes [34,35,36,37,38,39,40,41,42,43]. Hsp110s are one of the key players in maintaining protein homeostasis (proteostasis) [34,35,36,37,38,39,40,41,42,43,44,45,46,47,48,49,50,51,52,53,54,55,56,57,58,59,60,61,62,63,64,65,66,67,68,69], one of the most fundamental cellular processes for every living organism. Hsp110s act as indispensable molecular chaperones, playing dual roles as both independent chaperones and cochaperones for Hsp70s. Notably, Hsp110s exhibit a distinctive and pronounced chaperone activity in preventing the aggregation of denatured proteins, commonly referred to as holdase activity. The relatively low sequence conservation between human and fungal Hsp110s suggests that fungal Hsp110 may serve as a favorable target for designing novel and effective therapeutics for fungal infections. As the only Hsp110 in *C. albicans*, Msi3 is essential for the growth, survival, and infection of *C. albicans* in humans [35,70,71]. In contrast, knockout experiments in mice suggest that mammalian Hsp110s are not essential for normal development and growth [72,73,74]. Thus, we hypothesize that fungal Hsp110s, such as Msi3, represent a promising target for the design of novel and efficient antifungal medications with potentially low side effects. Recently, our laboratory characterized the biochemical properties of Msi3 and identified 2H, a pyrazolo [3,4-b] pyridine derivative, as an effective inhibitor of the biochemical and chaperone activities of Msi3 [33,75,76]. Importantly, 2H effectively inhibits the growth and viability of *C. albicans* [33]. Additionally, the fungicidal effect of 2H is linked to its inhibition of in vivo protein folding. Hsp110 genes were predicted in the available genomic sequences of *Candida* species and *C. neoformans*. Although these Hsp110s are not characterized yet, the high sequence conservation among fungal Hsp110s suggests that 2H may have a broad-spectrum antifungal activity.

In this study, further investigation of 2H and fungal Hsp110s revealed its expansive antifungal efficacy, effectively restraining the proliferation of *C. neoformans* while also perturbing the in vitro chaperone activity of Hsp110 from *C. auris*. Pharmacokinetic evaluations suggest that oral administration of 2H may offer superior therapeutic outcomes compared to intravenous delivery, underscoring the importance of optimizing the AUC/MIC ratio for advancing its clinical utility. Our objective is to enhance the potency and specificity of 2H for more effective fungal infection treatment.

## 2. Materials and Methods

### 2.1. Protein Expression and Purification

The expression and purification of auHsp110 followed the established protocols in our lab [33,75,76]. The ORF of the full-length auHsp110 (702 a.a.) was amplified from the genomic DNA of *C. auris* (generously provided by Dr. Theodore White, University of Missouri-Kansas City) and cloned into the pSMT3 vector. After the DNA sequence was confirmed by sequencing, the resulting pSMT3-auHsp110 plasmid was transformed into Novagen’s Rosetta2(DE3)pLysS strain (MilliporeSigma, Darmstadt, Germany) for expression. Induction of expression was carried at 18 °C for 6–8 h in LB Miller medium after adding IPTG to a final concentration of 1 mM. The auHsp110 was expressed as an Smt3 fusion protein with a His6 tag at the N-terminus. A HisTrap column (Cytiva, Uppsala, Sweden) was used for the first step of purification. Buffers containing 25 mM Hepes-NaOH, pH 7.5, 300 mM NaCl, 10% glycerol, and 1 mM TCEP were used. After incubating with the Ulp1 protease overnight, the Smt3 tag was cleaved off. A second HisTrap was applied to remove the Smt3 tag. The resulting protein was further purified on a HiTrap Q column (Cytiva, Uppsala, Sweden) using 25 mM Hepes-NaOH, pH7.5, as a buffer. Peak fractions were pooled, concentrated to >10 mg/mL, flash-frozen, and stored in a −80 °C freezer.

The firefly luciferase was expressed and purified as published before [33,75,76]. Briefly, the pSMT3-luciferase plasmid was transformed into BL21(DE3) and luciferase was expressed as an Smt3 fusion in a similar fashion as auHsp110. The expression was carried out at 30 °C for 4–6 h in LB Miller medium with IPTG at a final concentration of 1 mM. The Smt3-luciferase fusion protein was first purified on a HisTrap column. After the Smt3 tag was cleaved off by the Ulp1 protease, a second HisTrap column was used to separate the Smt3 tag from the luciferase. The resulting luciferase protein was concentrated to >10 mg/mL before flash freezing and storing at −80 °C.

### 2.2. Preventing Aggregation Assay for Analyzing the Holdase Activity of Hsp110

This assay was conducted following previously established protocols using purified luciferase as a model substrate [33,45,75,76,77]. Upon incubation at 42 °C, the purified luciferase aggregates over the time of incubation. The aggregation of purified luciferase protein was monitored using OD at 320 nm based on the light scattering of protein aggregates. The assay was carried out in 1 mL buffer A (25 mM MES, pH 6.5, 150 mM KCl, 2 mM Mg(OAc)_2_, 3 mM ATP, and 1 mM DTT). To analyze the holdase activity of auHsp110, auHsp110 was mixed with luciferase in the reactions. Prior to the assay, 4 μM of auHsp110 was incubated with either 2H (30 μM) or DMSO (solvent control) for 1 h at room temperature in buffer A. The final concentrations of luciferase, auHsp110, and 2H were 1 μM, 4 μM, and 30 μM, respectively.

### 2.3. Synergy Test on Candida Albicans

The assay was carried out as published previously [33,78,79]. SC5314, the most widely-used wild-type *C. albicans* strain, was obtained from Dr. Ronda Rolfes (Georgetown University). An aliquot of SC5314 was thawed and grown overnight in Sabouraud medium at 30 °C. After harvesting by centrifugation at 1200× *g* for 5 min, the cells were washed twice using PBS. Afterward, the inoculum was prepared in the YNB medium, a yeast synthetic medium, with an OD600 of 0.0005 (corresponding to 5 × 10^3^–1 × 10^4^ colony forming units (CFU)). Five common antifungals (Amphotericin B, Anidulafungin, Flucytosine, Clotrimazole, and Itraconazole) were prepared in either DMSO or ddH_2_O according to the manufacturer’s instructions. The antifungal drugs were serially diluted in DMSO or ddH_2_O and added to wells containing 100 µL inoculum in sterile 96-well culture plates. In addition, serial dilutions of 2H were added in combination with the above antifungals. Wells without any compounds were used as growth controls. Incubation at 37 °C followed, and optical density measurements at 600 nm were taken after 24 h with wells containing only medium as blanks. The fractional inhibitory concentration index (FICI) was calculated to test the synergistic effect between each of these common antifungals with 2H according to the published protocols [78,79].

### 2.4. Antifungal Susceptibility and Viability Testing on Cryptococcus Neoformans Type Strain H99

We followed the Clinical and Laboratory Standards Institute for yeasts (CLSI M27 [80]) and our previously published protocol for the determination of MIC and MFC [33]. The *C. neoformans* Type Strain H99 was obtained from the laboratory of Dr. Joseph Heitman (Duke University). After growing in Sabouraud medium, this strain was resuspended in 35% glycerol, aliquoted, flash-frozen in liquid nitrogen, and stored in a −80 °C freezer.

For each experiment, a 100 µL frozen aliquot of H99 was thawed and diluted into 5 mL Sabouraud medium. After being grown at 30 °C overnight, the culture was pelleted and washed twice using PBS at room temperature. The inoculum was prepared using the YNB medium, a yeast synthetic medium, with 5 × 10^3^–1 × 10^4^ colony forming units (CFU) (corresponding to an OD600 of 0.0005). Sterile 96-well culture plates were used for growth tests. After aliquoting 100 µL of the inoculum into each well, serial dilutions of 2H or fluconazole were added to the wells and mixed well by pipetting up and down. After shaking at 37 °C for 48 h, growth was measured by absorbance at 600 nm using a spectrophotometric plate reader. Wells with only medium were used as blanks and wells with only inoculum were used as growth controls. A viability test to determine the minimum fungicidal concentration (MFC) involved plating aliquots from wells with no observable growth on plates containing Sabouraud agar, followed by colony counting after incubation.

### 2.5. Pharmacokinetics Assay

All animal studies were performed following the animal protocol approved by the Institutional Animal Care and Use Committee of Shenzhen Bay Laboratory. 2H was dissolved in a solution containing 5% DMSO, 10% solutol, and 85% PBS. The concentrations of 2H were adjusted for IV (1 mg/mL) and PO (2 mg/mL) administrations. Male Institute of Cancer Research (ICR) mice (SPF grade, aged 6–8 weeks) were carefully selected to ensure uniformity (within 20% of weight variation). A fasting period of 12–16 h preceded the drug administration to minimize the impact of food. The selected animals were individually weighed, numbered, and then randomly grouped. The body weights were 38.5–42.9 g. The injection volume (V) was calculated based on the weight of each experimental animal using the formula: V (μL) = Weight (g) × Dose (mg/kg) ÷ Concentration (mg/mL). Following one dose of the drug administration, blood samples (150 µL) were collected via retro-orbital bleeding and placed into pre-heparinized microcentrifuge tubes at preset time points (5 min, 30 min, 1 h, 2 h, 4 h, 8 h, and 24 h). The blood samples were then centrifuged at 2500× *g* for 10 min to remove cells. The resulting plasma was transferred into 1.5 mL microcentrifuge tubes, which were subsequently frozen for analyzing 2H concentrations. After completion of blood collection, the mice were observed for two weeks before being euthanized. After precipitating plasma proteins using a mixed solution of acetonitrile/H_2_O at 3/1 ratio, the plasma samples were extracted. High-performance liquid chromatography (HPLC) analysis was then performed using an InfinityLab Poroshell 120 EC-C18 column (Agilent, Santa Clara, CA, USA) with a gradient elution of water and acetonitrile. The unique high UV absorbance of 2H allows for HPLC detection sensitive enough to measure concentrations as low as 10 ng/mL, eliminating the need for LCMS, which is more costly than HPLC. The concentrations of 2H were calculated using a calibration curve of 2H. The equations used to calculate the pharmacokinetic parameters are as follows:

Half-life: $t_{1/2}=Ln(2)/k_{e\lim ination}$;

Bioavailability: $F(\%)=\frac{AUC_{PO}/Dose}{AUC_{IV}/Dose}\times 100\%$;

Area under the curve: $AUC=\int_{0}^{t}Cdt$;

Mean residence time: $MRT=\int_{0}^{t}t•Cdt/\int_{0}^{t}Cdt$;

Clearance: CL = dose/AUC;

Volume of distribution: Vss = CL × MRT.

## 3. Results

### 3.1. No Apparent Synergy between 2H and Several Common Antifungal Drugs

Different from the five known classes of available antifungal drugs, the inhibition of 2H on *C. albicans* represents a novel mode of action. It is possible there is synergistic effect between 2H and some of the available antifungals. Consistent with this hypothesis, it has been proposed that targeting stress responses, such as those involving heat shock proteins, would be a promising strategy to enhance the efficacy of established antifungals [81,82]. Synergistic antifungal combination is a promising strategy in treating fungal infections with multiple benefits such as being more effective than single antifungals, overcoming resistance, and allowing for the use of lower doses of antifungals, which can reduce concerns of toxicity and cost of some antifungals [83,84]. To explore potential synergistic effects, we examined five common, approved antifungals when co-administered with 2H: Amphotericin B, Anidulafungin, Flucytosine, Clotrimazole, and Itraconazole. These antifungal agents belong to three classes of antifungals (azoles, polyenes, and echinocandins), as well as Flucytosine [20,21,22,25,26,27]. Amphotericin B, a polyene antifungal, disrupts fungal cell membrane integrity by forming pores by interacting with ergosterol, leading to the leakage of cellular components and subsequent fungal cell death [85]. Anidulafungin [86], an echinocandin, inhibits fungal cell wall synthesis by targeting the enzyme beta-(1,3)-D-glucan synthase. Flucytosine [27,87], an antimetabolite and toxic pyrimidine analog, converts into 5-fluorouracil within the fungal cells, interfering with RNA synthesis and causing cell death. Clotrimazole [88] and itraconazole [89], both azole antifungals, inhibit the synthesis of ergosterol, a crucial component of fungal cell membranes. There are three types of azoles based on the number of nitrogen atoms on the five-membered ring: imidazoles, triazoles, and tetrazoles [90,91]. Clotrimazole is imidazole while itraconazole and fluconazole belong to triazoles. Our previous test yielded no apparent synergistic effect between 2H and fluconazole [33]. Thus, fluconazole was not included in this test.

**Figure 1:**
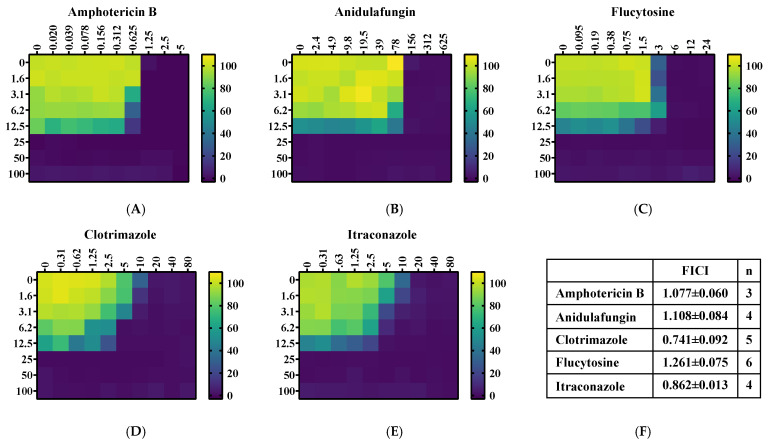
Synergy testing between 2H and several common antifungal drugs (**A**–**E**); Checkerboard analysis of antifungal activity for combination of 2H and several common antifungals. The heat-map plots of relative growth (calculated by setting the growth without compounds as 100%) were presented: (**A**) Amphotericin B; (**B**) Anidulafungin; (**C**) Flucytosine; (**D**) Clotrimazole; (**E**) Itraconazole. Color scale for relative growth is provided on the right. (**F**) The Fractional Inhibitory Concentration Index (FICI) for the combination of 2H and several common antifungals. The FICI is interpreted as follows: a synergistic effect when FICI ≤ 0.5; an additive or indifferent effect when FICI > 0.5 and <2; an antagonistic effect when FICI > 2.

A checkerboard (or chequerboard) assay was carried out to assess the fractional inhibitory concentration index (FICI). The widely-used wild-type *C. albicans* strain SC5314 was used. As shown in Figure 1, the FICI values for all the five tested antifungals were in the ranges of 0.5–2, suggesting the absence of obvious synergy between 2H and the evaluated antifungal agents. More antifungals may need to be examined to find potential synergistic effects with 2H.

### 3.2. 2H Abolishes the Chaperone Activity of the Hsp110 from Candida Auris

In addition to *C. albicans*, several *Candida* species can cause serious infections in humans including *C. auris* and *C. glabrata*. Previously, we have shown that 2H efficiently inhibits the chaperone activity of Msi3 as well as the growth and viability of several fungal species including *C. albicans*, *C. glabrata*, and *S. cerevisiae* [33]. There is high sequence conservation in Hsp110s among *Candida* species (Figure S1). Thus, we hypothesize that 2H will inhibit the growth and viability of many *Candida* species through inhibiting the chaperone activity of their Hsp110s.

**Figure 2:**
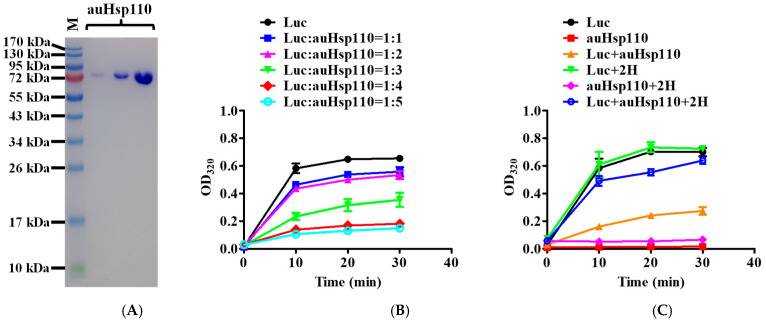
2H inhibits the holdase activity of purified Hsp110 from *C. auris* (auHsp110). (**A**) auHsp110 was purified to high purity. Various amounts of purified auHsp110 (0.3, 1, and 3 µg) were loaded on an SDS-PAGE. Protein marker (M) is shown on the left. (**B**) The purified auHsp110 showed a strong holdase activity in preventing the aggregation of firefly luciferase (Luc). The preventing aggregation activity was assayed based on the OD reading of Luc at 320 nm after incubating at 42 °C. An increased OD reading represents the aggregation of Luc. Different ratios of Luc/auHsp110 were tested and listed on the right of the plot. The plots represent averages with SEM (n = 4), using two different protein purifications. (**C**) The holdase activity of auHsp110 was eliminated after incubating with 2H. The holdase activity of auHsp110 was assayed in the same way as in (B), using Luc as a model substrate. The Luc/auHsp110 ratio was 1:4 and the concentration of 2H was 30 µM. Each plot was averages with SEM from three independent experiments (n = 3) using two different protein purifications.

*C. auris* represents an emerging global health threat due to its multidrug resistance. According to the genomic sequence, there is one hypothetical Hsp110 in *C. auris*, which we named auHsp110. Based on the high sequence conservation between auHsp110 and Msi3 (Figures S1 and S2), we hypothesize that 2H inhibits the chaperone activity of auHsp110 and growth of *C. auris*. To test this hypothesis, we expressed auHsp110 and purified it to high purity (Figure 2A). To analyze the effect of 2H on the chaperone activity of auHsp110, we first tested the holdase activity of auHsp110 in preventing protein aggregation. To analyze the holdase activity, we took advantage of the well-established assay using purified luciferase as a model substrate. In this assay, upon heating to 42 °C, luciferase denatures and aggregates, which shows an increased OD reading at 320 nm due to the enhanced light scattering of the luciferase aggregates (Figure 2B, black circles). Adding auHsp110 during heating effectively reduced the aggregation of luciferase in a concentration-dependent manner (Figure 2B). Thus, auHsp110 demonstrates a similar holdase activity as reported previously for Msi3 and human Hsp110 (hHsp110) in preventing the aggregation of luciferase [33].

At a 1:4 ratio of luciferase to auHsp110, the holdase activity approaches its maximum (Figure 2B, red diamond). We then used this ratio to test the impact of 2H on the holdase activity of auHsp110. Importantly, incubating with 2H effectively inhibited the in vitro holdase activity of auHsp110 in a similar fashion as observed for Msi3 (Figure 2C), providing support for our hypothesis. Since this inhibition on the holdase activity of Hsp110 is directly related to the fungicidal activity of 2H, 2H most likely will have a similar antifungal effect on *C. auris*. Due to the limitation of biosafety, we are not able to test the effect of 2H on *C. auris* directly. Together with the inhibition on *C. albicans* and *C. glabrata* [33], 2H most likely represents a new class of a broad-spectrum antifungal for *Candida* species.

### 3.3. 2H Inhibits Growth of Cryptococcus Neoformans and Is Fungicidal

We next tested the fungicidal spectrum of 2H against non-*Candida* fungal pathogens to determine 2H’s potential for broad-spectrum activity. *Cryptococcus neoformans* is another common class of important fungal pathogen other than *Candida* species. There is one predicted Hsp110 in *C. neoformans* (cnHsp110). Although the sequence conservations between *C. albicans* and *C. neoformans* are lower than those within the *Candida* species, the conservations are still higher than that between Msi3 and human Hsp110 (Figures S1 and S2). Moreover, fungal Hsp110s share a similar molecular weight (~80 kDa), which is much smaller than that of human Hsp110 (~110 kDa), suggesting functional conservation among fungal Hsp110s. Thus, it is possible that 2H is still effective in inhibiting these fungal pathogens.

**Figure 3:**
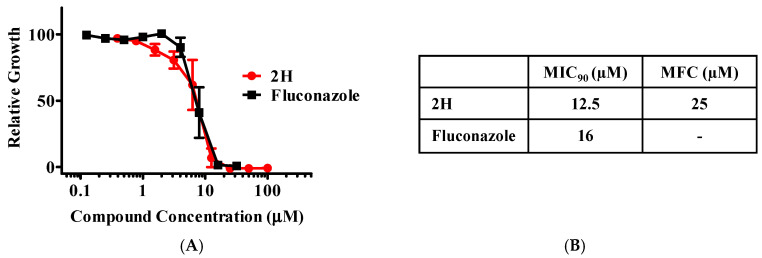
2H inhibits the growth of *Cryptococcus neoformans* and is fungicidal. (**A**) The effect of 2H on the growth of *C. neoformans* strain H99. Fluconazole was used as a control. Relative growth was plotted by setting the growth in the absence of compound as 100%. Data are presented as mean values +/− SEM (n = 4 independent experiments). (**B**) The MIC_90_ and MFC of 2H were determined on *C. neoformans* strain H99. MIC_90_: the minimum inhibitory concentration for 90% inhibition. The minimal fungicidal concentration (MFC): the minimal concentration of drug resulting in less than 0.1% of the cells alive relative to the original inoculum. Fluconazole was used as a control.

To evaluate the effect of 2H on *C. neoformans*, we carried out growth tests on the widely-used H99 strain. As shown in Figure 3A,B, 2H efficiently inhibited the growth of H99 with an MIC_90_ (minimum inhibitory concentration for 90% inhibition) of 12.5 µM. Notably, the MIC_90_ of 2H surpassed the efficacy of fluconazole when confronting the H99 strain. Fluconazole is a common approved antifungal. Based on whether fungal pathogens are killed, antifungals are divided into two classes: fungistatic (inhibits growth only) and fungicidal (kills the pathogen). Azoles such as fluconazole and itraconazole are normally fungistatic. In general, resistance develops faster to fungistatic antifungals than to fungicidal agents after prolonged usage. Consistent with the fungicidal effect on *C. albicans*, 2H was fungicidal towards *C. neoformans* with an MFC (minimum fungicidal concentration, the minimum concentration for killing > 99.9% cells) of 25 μM (Figure 3B). In contrast, the fluconazole control was noted for being fungistatic. Both the MIC_90_ and MFC of 2H toward H99 were similar to those toward *C. albicans*, suggesting 2H has a similar antifungal activity on this species of common fungal pathogen.

Taken together, the findings indicate that 2H possesses strong antifungal properties, exhibiting effectiveness against a wide range of fungi including *Candida* species and *C. neoformans.*

### 3.4. Pharmacokinetics Profiles of 2H after IV and PO Administrations to ICR Mouse

**Figure 4:**
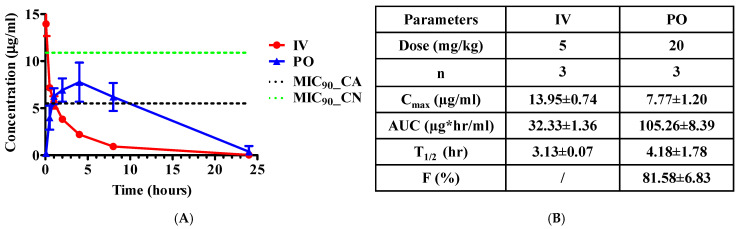
Pharmacokinetic profiles of 2H after administrations to mice. (**A**) The plasma concentrations of 2H after intravenous injection (IV) and taken through the mouth (PO stands for per os). Institute of Cancer Research (ICR) mice were used. The doses for IV and PO were 5 mg/kg and 20 mg/kg, respectively. The plots are presented as mean values ± SEM (n = 3 animals). The dotted lines indicate the MIC_90_ values for *C. albicans* SC5314 (green) and *C. neoformans* H99 (black). (**B**) The PK parameters derived from (**A**). n, the number of mice used; C_max_, the maximum concentration of 2H, i.e., the peak level; AUC, area under the concentration time curve; T_1/2_, half-time; F, bioavailability. The reported values are mean ± SEM.

To assess the potential of 2H as an antifungal for clinical use, we carried out an analysis to determine the pharmacokinetic (PK) profile of 2H. The Institute of Cancer Research (ICR) mouse is the most commonly used outbred normal mouse strain for PK studies. We chose this strain for our PK studies due to its excellent reproductive performance, cost-effectiveness, and robustness. Both intravenous (IV, at 5 mg/kg) and per os (PO, at 20 mg/kg) administrations were conducted (Figure 4). Typically, a dosage range of 1–10 mg/kg is recommended for IV administration and 10–50 mg/kg for PO administration. These ranges are high enough to be detected in plasma and tissue, but low enough to be safe and not cause any major toxicity. The PO dosage is relatively higher than that of the IV dosage due to differences in absorption and bioavailability. Our data suggest that these dosages of 2H are well tolerated and reasonable for both detection and safety. The IV administration yielded a maximum plasma concentration (C_max_) of 13.95 ± 0.74 μg/mL right after administration; but a quick reduction in plasma concentration was observed. At 1 h after administration, the plasma concentration was down to 5.62 ± 0.38 μg/mL, a more than two-fold reduction from the initial concentration. At 8 h, the plasma concentration was below 1 μg/mL. The PO delivery led to a C_max_ of 7.77 ± 1.20 μg/mL at 4 h (Figure 4). Although the C_max_ for PO administration was lower than that of IV administration, the plasma concentration remained close to C_max_ from 1 h to 8 h after administration (Figure 4A), relatively higher than that of PO administration. Impressively, the bioavailability for PO was >80% (Figure 4B), underscoring the efficiency of absorption and systemic availability of 2H through the oral route.

The MIC_90_ and MFC values of 2H for various fungal species determined by our previous and current analyses range from 5.5 μg/mL (12.5 µM) to 10.9 μg/mL (25 µM) [33]. Thus, the AUC/MIC_90_ ratios for 2H range from 3 to 19 (Figure 4B, AUC: area under the concentration time curve). Notably, for *C. neoformans* (Figure 3) and the fluconazole-resistant strain FH5 of *C. albicans* tested in our previous study [33], the MIC_90_ was 5.5 μg/mL (12.5 µM) and the AUC_PO_/MIC_90_ ratio reached to 19.3 (Figure 4B), indicating the possibility for clinical therapy. In addition, none of the mice exhibited discernible signs of toxicity throughout the two week observation period following 2H treatment. This observation is noteworthy, suggesting a certain level of safety and tolerability for the administered doses.

## 4. Discussion

The compound 2H has emerged as a promising lead for the development of novel antifungals with a new mode of action. The currently approved antifungals are categorized into five classes: azoles, polyenes, echinocandins, allylamine, and triterpenoids [20,21,22,23,24,25,26], primarily targeting the cell membrane and cell wall. Different from these approved antifungals, 2H targets Hsp110, an essential chaperone for maintaining cellular protein homeostasis. This novel mechanism provides a new option to combat the emerging resistance against the currently approved antifungals.

The combination of two or more antifungals with synergistic effects has proven more effective in treating fungal infections than a single antifungal [83,84]. However, while testing representative antifungals from four approved class of antifungals alongside 2H, no synergistic effect was observed. A large-scale screen on more antifungals may be important for searching antifungals that show synergy with 2H.

PK analysis revealed an AUC/MIC_90_ range of 3 to 19 for 2H, suggesting the potential of 2H for clinical therapy, especially for *C. neoformans* and the fluconazole-resistant strain FH5 of *C. albicans* with MIC_90_ at 5.5 μg/mL (12.5 µM). The C_max_ for IV and PO administrations were 13.95 ± 0.74 μg/mL and 7.77 ± 1.20 μg/mL, respectively. Based on our previous and current analysis on several pathogenic fungal species, including four fluconazole-resistant strains of *C. albicans* [33], the MIC_90_ and MFC values of 2H were between 5.5 μg/mL (12.5 µM) and 10.9 μg/mL (25 µM) [33]. Thus, the C_max_ value for IV is well above the MIC_90_ and MFC for all the fungal species that were evaluated. However, the fast reduction of plasma concentration (i.e., short T_1/2_) makes IV administration less promising for clinical usage. In contrast, the plasma concentration of 2H stayed close to C_max_ for more than 7 h following PO delivery, making it more desirable for clinical administration. Although the C_max_ for PO is well above the MIC_90_ of *C. neoformans* and FH5 (5.5 μg/mL), it is lower than the MIC_90_ and MFC values for all the remaining strains of *Candida* tested. Improving the C_max_ for PO delivery or T_1/2_ for IV administration is important for advancing 2H for clinical therapy. To achieve plasma concentrations above the MIC_90_ values of all the tested *Candida* species, future PK analyses with higher dosages may be conducted if no apparent toxicity is observed. Additionally, 2H demonstrates favorable safety and oral bioavailability profiles. However, for a comprehensive evaluation of efficacy, safety, and tolerability, pharmacodynamic assessments, such as mouse models of fungal infection, are imperative before progressing to clinical trials.

*C. auris* is a multidrug-resistant pathogen that poses significant global health threats. Our investigation revealed that 2H effectively inhibits the holdase activity of auHsp110. This finding suggests that 2H holds promise as a therapeutic agent for the treatment of *C. auris* infections and emerges as a compelling candidate to address the challenge of multidrug resistance in this pathogen. Future antifungal tests on *C. auris* will confirm the antifungal effect of 2H.

Together with our previous studies, we have shown that 2H is fungicidal for three fungal species: *C. albicans*, *C. glabrata*, and *C. neoformans*. In addition, our published work has shown that four strains of fluconazole-resistant strains of *C. albicans* are inhibited by 2H in a similar fashion [33,92,93,94]. Notably, *C. albicans* FH5 [33,93] and *C. neoformans* may exhibit higher susceptibility to 2H compared to other *Candida* species. The inhibition of auHsp110’s chaperone activity by 2H suggests the potential of 2H in inhibiting and killing *C. auris*. Taken together, 2H may represent a new class of a broad-spectrum antifungal and holds promise as a potent candidate for the development of antifungal therapies.

To date, a comprehensive complex structure involving fungal Hsp110s and 2H has yet to be elucidated, rendering the precise binding sites unresolved. Our data suggest that 2H predominantly binds to the substrate-binding domain (SBD) of Hsp110 [33]. Therefore, understanding the distinctions between human Hsp110 and fungal Hsp110s, particularly in the SBD, is essential for the design of specific and potent antifungals. Utilizing structure-guided approaches for drug refinement holds promise in enhancing both precision and efficacy. Furthermore, optimizing the properties of 2H through structure-guided approaches stands as the most promising strategy for advancing its therapeutic potential, such as improving solubility, permeability to fungal pathogens, and PK profiles as described above. Once we obtain improved compounds, we will carry out comprehensive multiple dosing PK analyses to determine the accumulation and toxicity of these compounds and evaluate their clinical relevance.

## Data Availability

The original contributions presented in the study are included in the article/Supplementary Materials, further inquiries can be directed to the corresponding author.

## Supplementary Materials

The following supporting information can be downloaded at: https://www.mdpi.com/article/10.3390/jof10110732/s1, Figure S1: The pairwise sequence identifies among selective Hsp110s; Figure S2: Sequence alignment of selective Hsp110s.
