# Supplementary material for: Characterization of a New Hsp110 Inhibitor as a Potential Antifungal
Source: J Fungi (Basel). 2024 Oct 23;10(11):732. doi: 10.3390/jof10110732 (PMC11595998; doi:10.3390/jof10110732)
Supplement: Supplementary file 1 [file jof-10-00732-s001.zip › jof-3194541-supplementary.pdf]

Figure S1

	Msi3 (701 a.a.)	hHsp110	Length (a.a.)
**hHsp110**	**38.5**	**-**	**858**
**Apg-1**	**36.7**	**59.2**	**839**
**Apg-2**	**37.5**	**64.28**	**840**
**Hsp110-*auris***	**73.3**	**37.7**	**702**
**Hsp110-*glabrata***	**63.7**	**39.2**	**694**
**Hsp110-*parapsilosis***	**83.9**	**38.2**	**706**
**Hsp110-*tropicalis***	**88.7**	**39.5**	**691**
**Hsp110-*Cryptococcus***	**45.9**	**41.9**	**773**

Figure S1 – The pairwise sequence identifies among selective Hsp110s. hHsp110, Apg-1 and Apg-2 are the three known human Hsp110s. Hsp110-*auris,* Hsp110-*glabrata,* Hsp110-*parapsilosis,* and Hsp110-*tropicalis* are the hypothetical Hsp110s from four common pathogenic *Candida* species *C. auris, C. glabrata, C. parapsilosis, and C. tropicalis,* respectively. Hsp110-*Cryptococcus* is the Hsp110 predicted from the genomic DNA sequence of *Cryptococcus neoformans*.

Figure S2

```
                         1         10        20        30        40        50
hHsp110                .MSVVGLDVGSQSCYIAVARAGGIETIANEFSDRCTPSVISFGSKNRTIGVAAKNQQITH
Msi3                   MSIPFGVDLGNNNTVIACAKNRGIDIVVNEVSNRSTPSLVGFGHKSRYLGESAKNQQTSN
Hsp110-auris           MATPFGVDFGNDNTVIACARNRGIDIIVNEVSNRSTPSLVGFGQRNRFIGETGKNQQTSN
Hsp110-Cryptococcus    MASVVGIDLGNLSSKVGVARHRGIDIIVNEVSNRATPSLVSFTPRQRFIGESAKTAETSN

                       60        70        80        90        100       110
hHsp110                ANNTVSNFKRFHGRAFNDPFIQKEKEN.LSYDLV.PLKNGGVGIKVMYMGEEHLFSVEQI
Msi3                   IKNTVDNLKRIVGLPHDHPDFEIEKK.FFTIPLIKNEVDGGVSGKVKYLTKNHEFTATQL
Hsp110-auris           LKNTVGNLKRILGMNYKDPDFDIESK.YFSCGLGENEL.GQINAKVRFLNEPQEFTSTQL
Hsp110-Cryptococcus    FKNTVGSLKRLIGRSFNDPEVEEYEKKFINAQL..VDVNGEIGVKVNYLGEPTDFSFTQL

                       120       130       140       150       160       170
hHsp110                TAMLLTKLKETAENSLKKPVTDCVISVPSFFTDAERRSVLDAAQIVGLNCLRLMNDMTAV
Msi3                   LAMYLDKIKDTALKETKGNISDICLSVPGWYTEKQRRAAADACKIAGLNPVRIVNEVTAA
Hsp110-auris           AAMFLNKIKDITAKETKGNIVDICLSVPVWYTEKQRRAASDACKIAGLNPVRIVNEVTAA
Hsp110-Cryptococcus    VAAYLGKLRDTTAAELKQSVSDVVIAVPGWFTDVQRRALLDAANIAGLNALRLINDNTAV

                       180       190       200       210       220       230
hHsp110                ALNYGIYKQDLPSLDEKPRIVVFVDMGHSAFQVSACAFNKGKLKVLGTAFDPFLGGKNFD
Msi3                   AVGYGVFKAGEL.PEDEYKKVAFVDVGHSSYQVSIAAVKKGELKILGSAYDKHFGGRNFD
Hsp110-auris           AVGYGVFKQNEL.PEDKPKNVAFVDIGHSSFQVSIAAVKKGELKILGSAYDKHFGGRDFD
Hsp110-Cryptococcus    ALGYGITKADLPESTEAPRHVVFVDVGHSDYSVAVVAFSKGQLTIKSTAYDRHFGGRDFD

                       240       250       260       270       280       290
hHsp110                EKLVEHFCAEFKTKYKLDAKSKIRALLRLYQECEKLKKLMSSNSTDLPLNIECFMNDKDV
Msi3                   FAIAEHFAKEFKSKYKIDVHENPKAFYRVLVAAEKLKKVLSAN.TQAPFNIESVMNDVDV
Hsp110-auris           RAITEHFADEFKTKYKIDIRENPKAYYRVLTAAEKLKKVLSAN.TQAPINIESVMNDIDV
Hsp110-Cryptococcus    YALVQHFAEEFKTKYKIDVLSSPKAVFRLTTGCERLKKVLSAN.TEAPINVESLMNDIDA

                       300       310       320       330       340       350
hHsp110                SGKMNRSQFEELCAELLQKIEVPLYSLLEQTHLKVEDVSAVEIVGGATRIPAVKERIAKF
Msi3                   SSSLTREELEELVQPLLDRINVPIETALKDAGITVDELDSIEVIGGSSRIPAVKTRISEI
Hsp110-auris           SSSLTREDLEEFIKPLLERINVPVEIALKDAGLTTEQIDSIEIIGGCTRVPSLKKRLTEI
Hsp110-Cryptococcus    TSTLTRESFEKLTDHLLSRVSVPLAEALEKAGLTIDQIDAVELVGGSTRIPAIKERIQQF

                         360       370       380       390       400       410
hHsp110                FG.KDISTTLNADEAVARGCALQCAILSPAFKVREFSVTDAVPFPISLIWNHDSED..TE
Msi3                   FG.KPLSFTLNQDEAIAKGNAYICACHSPTVRVRPFKFEDYNQYTVSYFWDKEDE...ED
Hsp110-auris           FG.KPLSTTLNQDEAIARGNAFICAMHSPTLRVRPFKFEDFNPYSVSYFWDKEEE...DD
Hsp110-Cryptococcus    FGGKVLNFTLNQDEAIARGATFACASLSPVFRVREFAVHDIAAYPIKISWEKEAGNPDED

                           420       430       440       450       460       470
hHsp110                GVHEVFSRNHAAPFSKVLTFLRRG.PFELEAFYSDPQGVPYP.EAKIGRFVVQNVSAQKD
Msi3                   DHLEVFPKGGLFPSTKIITLFRKGPSFEIEAKYTKPEELPKGTELHIAKWKISGVVPNEG
Hsp110-auris           DHLEVFPRGGTFPSTKIITLFRKG.DFDVEARYTNKEELPAGVEPLIAKWHIKGVKPNEG
Hsp110-Cryptococcus    TELVVFGTANPIPSTKVLTFYRQG.AFELEAAYADPASLPKGINPWIGKYTVKSVEKPAS

                             480       490       500       510       520       530
hHsp110                GEKSRVKVKVRVNTHGIFTISTASMVEKVPTEENEMSSEADMECLNQRPPENPDTDKNVQ
Msi3                   ESSIATKIKLRNDPSGFYTIESAHTVEEQIVKELIE............PAEGEEVDE...
Hsp110-auris           ESSIATKLKLRNDPSGFYTIESAHTVEEKIVKELVE..........KEPKEGEEEEE...
Hsp110-Cryptococcus    GDLSIVKVKARLNLHGIMNFEGAYCVEEVEKEE................EVTVGEGE...

                             540       550       560       570       580       590
hHsp110                QDNSEAGTQPQVQTDAQQTSQSPPSPELTSEENKIPDADKANEKKVDQPPEAKKPKIKVV
Msi3                   ..............................................DAEPQYREVKKLVK
Hsp110-auris           ..............................................ESEPEYREVKKVVK
Hsp110-Cryptococcus    ..............................................DAKTEKKLVKKIQR
```

Figure S2

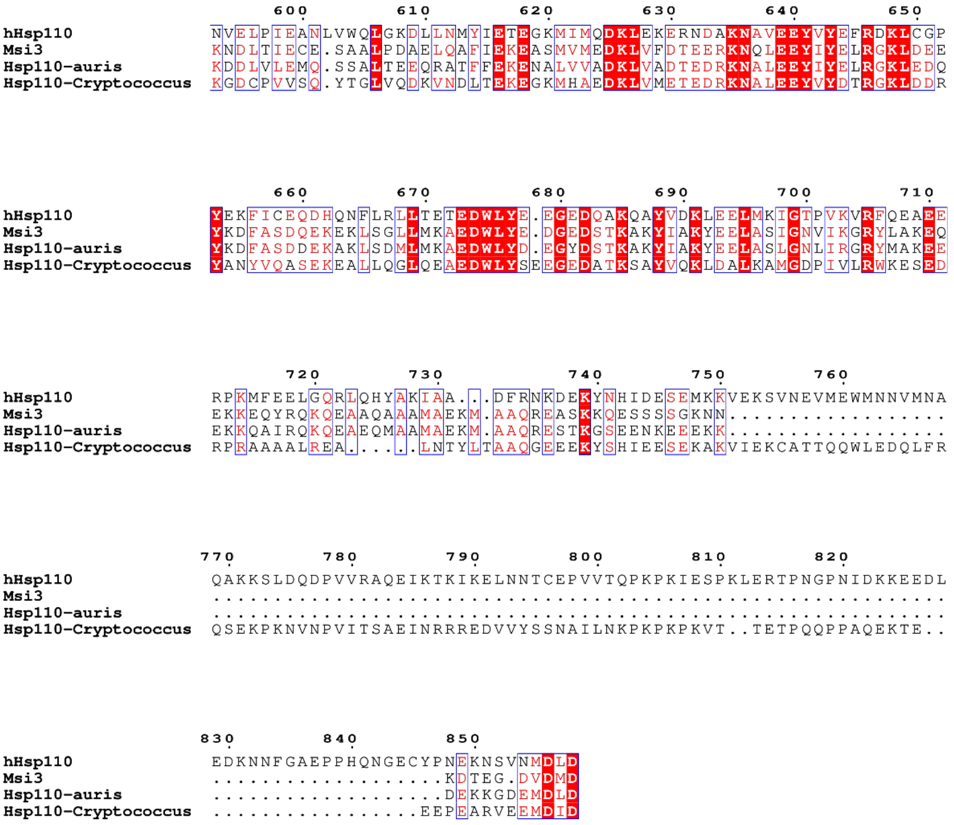

Figure S2 – Sequence alignment of selective Hsp110s.
hHsp110 is the major human Hsp110. Hsp110-*auris* and Hsp110- *Cryptococcus* are the hypothetical Hsp110s from *C. auris* and *Cryptococcus neoformans*, respectively, predicted from the genomic DNA sequences.
